# Promising advances in clinical trials of dental tissue-derived cell-based regenerative medicine

**DOI:** 10.1186/s13287-020-01683-x

**Published:** 2020-05-12

**Authors:** Yoichi Yamada, Sayaka Nakamura-Yamada, Ryutaro Konoki, Shunsuke Baba

**Affiliations:** 1 Department of Oral Implantology, Osaka Dental University, 1-5-17 Otemae Chuoku, Osaka, 540-0008 Japan; 2 Department of Infectious Diseases and Applied Immunology, IMSUT Hospital of The Institute of Medical Science, The University of Tokyo, 4-6-1 Shiroganedai, Minato-ku, Tokyo, 108-8639 Japan

**Keywords:** Stem cell therapy, Clinical application, Clinical trial, Regenerative medicine, Systematic review

## Abstract

**Background:**

Advances in regenerative medicine with stem cells have led to clinical trials. Dental/oral tissues are emerging as promising cellular sources of human mesenchymal stem cells. Recently, dental tissue-derived cells have been used clinically due to their great potential, easy accessibility, and ability to be obtained via methods with low invasiveness. The aim of this study is to systematically assess the clinical effectiveness of dental cell-mediated therapies compared to current evidence-based methods in human patients.

**Methods:**

The electronic databases MEDLINE, Cochrane Central Register of Controlled Trials (CENTRAL), and ClinicalTrials.gov were searched up to December 2019 for clinical trials. Clinical trials with any intervention using stem cells/cells derived from dental tissue were included.

**Results:**

A total of 815 studies were identified by the electronic search, and 38 articles qualified for full-text evaluation. Finally, 20 studies (10 clinical trials using dental pulp-derived cells, 3 clinical trials using periodontal ligament-derived cells, and 7 studies using gingiva-derived cells) were included in this review. No clinical trials using dental follicle- or apical papilla-derived cells were selected in this review. Dental pulp-derived cells were used in clinical trials for bone regeneration, periodontitis, and dental pulp regeneration. All clinical trials using periodontal ligament-derived cells and gingiva-derived cells were conducted for periodontal disease treatment and gingival augmentation, respectively. Among the 20 selected studies, 16 showed clinical benefits of cell transplantation therapies. In addition, no study reported adverse events that may have been associated with cell transplantation.

**Conclusions:**

These findings indicate that dental tissue-derived cells would be useful for cell-based regenerative medicine for various diseases.

## Background

Regenerative medicine has emerged as a novel therapeutic approach to promote regeneration in a more predictable manner. It is an attractive medical alternative to conventional treatment. Stem cells used as three elements (cells, scaffolds, and signaling molecules) are the most critical components for regeneration and play a pivotal role in tissue engineering and regenerative medicine. There are a variety of sources of stem cells, including pluripotent stem cells; embryonic stem (ES) cells, which are not widely used due to ethical concerns and the risk of tumor formation [1] and induced pluripotent stem (iPS) cells, which have the capacity to transform adult somatic cells back into pluripotent cells and the potential to form tumors [2]; and adult stem cells, for example, hematopoietic stem cells and mesenchymal stem cells (MSCs), which are multipotent, have the ability to self-renew, can be obtained from multiple sources, and are easy to access [3]. The latter cells have the greatest potential in tissue engineering for clinical application and were first identified in the bone marrow. However, researchers have been exploring other sources of MSCs because of the difficulty of harvesting a sufficient cell number and the pain and morbidity experienced during the harvesting procedure. Therefore, many anatomical locations have been investigated as sources of MSCs [4], and MSCs can currently be obtained from a number of tissues, such as adipose tissue [5], muscle, and dermis [6]. One of the potential sources of MSCs is dental/oral tissues containing dental pulp, periodontal ligaments, the oral mucosa, and the gingiva [7–10]. The use of MSCs of dental origin has increased exponentially in the last decade.

In dentistry, frequent clinical trials using regenerative medicine without cells have been conducted for a long time. Dentistry is at the forefront of regenerative therapy research, because it is in the craniofacial arena that many of these therapies would be first clinically applied. Biomaterials have traditionally been tested in the dental and craniofacial fields before being utilized in other medical specialties because the related areas are easy to access and are relatively smaller and have a lower load-bearing capacity than other anatomical locations [11]. The principal organ needing regeneration is the tooth, along with the surrounding hard and soft tissues, including periodontal tissue and craniofacial structures. Synthetic materials, growth factors, cytokines, biological extracellular matrices, and combinations of these components have been clinically used to reconstitute and restore the function of tissues and organs. However, due to the limited ability of these approaches, a cell-based approach has been developed. Recently, several preclinical animal studies regarding the use of adult stem cells have demonstrated promising results, and the stage has been precisely set for clinical application. Based on the numerous basic and pre-clinical studies, cell-based clinical trials just get to be performed [12–14]. Although there have been several reviews about preclinical studies using dental stem cells or clinical trials using biomaterials such as synthetic materials and growth factors, no systematic review of clinical trials using dental tissue-derived cells has been reported. Thus, the aim of the present study was to systemically review the current literature and to evaluate the current reality and feasibility of dental cell-based therapy in clinical applications.

## Methods

### Study design

This systematic review was conducted in accordance with the Cochrane Handbook for Systematic Reviews of Interventions and the Preferred Reporting Items for Systematic Reviews and Meta-analyses (PRISMA) guidelines. The focused “PICO” (Participants, Interventions, Comparisons and Outcomes) question was “In comparison to current conventional methods, are dental-derived stem cell/cell-based therapies effective in human patients?”

### Search strategy

The electronic databases MEDLINE (via PubMed), The Cochrane Central Register of Controlled Trials (CENTRAL), and ClinicalTrials.gov were searched for literature up to December 2019. The medical subject heading (MeSH) terms and keywords used for the search were “dental pulp stem cells,” “periodontal ligament cells,” “gingival fibroblast,” “gingival cells,” “dental follicle,” “apical papilla,” “Stem cells from human exfoliated deciduous teeth,” “DPSCs,” “SHED,” “PDL,” and “clinical study.”

### Inclusion criteria

(i) Clinical trials with a randomized controlled trial (RCT) design or a nonrandomized controlled trial (CT) design and descriptive studies (case series without a control group, controlled cohort studies).
(ii) Any intervention using stem cells or cells derived from dental tissue.

### Exclusion criteria

(i) In vitro or preclinical studies using human subjects.
(ii) Review articles.
(iii) Clinical studies that had insufficient data.
(iv) Clinical studies that were not completed.
(v) Non-English articles.

### Screening methods, data extraction, and quality assessment

Two reviewers performed the primary search by independently screening the titles and abstracts. The same reviewers evaluated the full manuscripts of studies meeting the inclusion criteria or with insufficient data in the title and abstract to make a clear decision. The reference lists of the selected studies were manually searched to find additional studies missed by the electronic searches. Differences in the assessment of eligibility were resolved by discussion. Data were extracted based on author names, the year of publication, the clinical trial registration ID, the condition, the study design, the number of patients and cases, interventions (test and control), follow-up periods, main outcomes, adverse events, and funding sources. Quality classification was assessed by using the revised Cochrane risk-of-bias tool for randomized trials (RoB 2) and ROBINS-I tool for nonrandomized trials. The domains included bias arising from randomization, deviations from the intended interventions, missing outcome data, measurement of the outcome, and selection of the reported results. The overall risk of bias was assessed by completing a “risk of bias” table for each included study.

## Results

### Search results

**Fig. 1:**
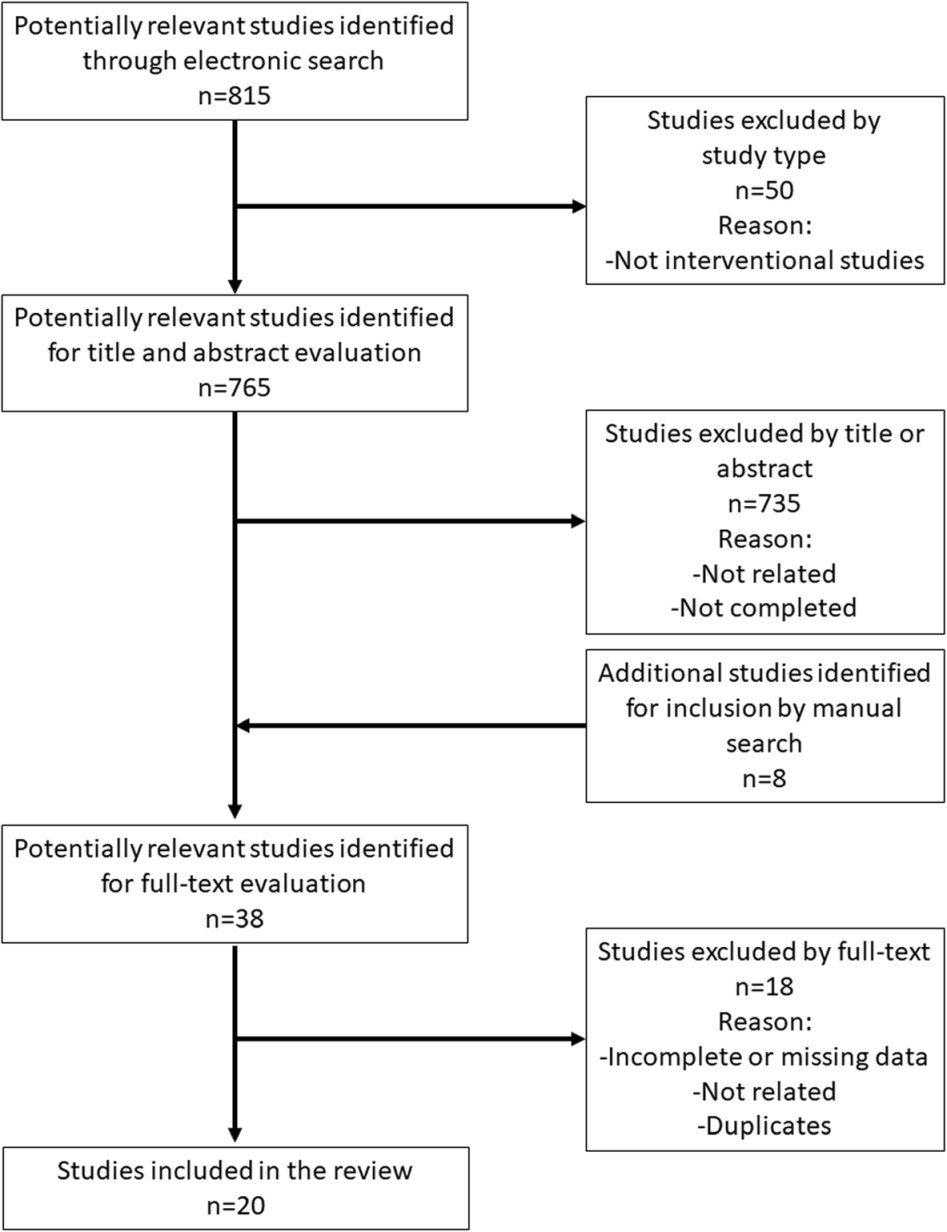
Flowchart for study selection (*n* = number of articles)

A flow chart of the search strategy is shown in Fig. 1. A total of 815 studies were identified by the electronic search. Of those studies, 765 were eligible for title and abstract screening by screening of the study type. After evaluation of the titles and abstracts, 735 studies were excluded because they did not meet the inclusion criteria. After the addition of 8 articles found in the manual search, 38 articles were qualified for full-text evaluation. Eighteen studies were excluded for the following reasons (Supplementary Table 1): Eight studies were excluded as identical clinical trials. Two studies were excluded because they were published protocols of clinical trials. Two studies were excluded because they did not use cells derived from dental tissues. Two studies were excluded because they had incomplete or missing data. Four studies were excluded because no results were available. Finally, 20 studies were included in this review. Because the selected studies showed heterogeneity, meta-analysis was not possible.

### Characteristics of the included studies

All studies except one (NCT01932164) were published between 2000 and 2018. Of the 20 selected studies, 10 used dental pulp-derived cells, 7 used gingiva-derived cells, and 3 used periodontal ligament-derived cells. No clinical trials using dental follicle- or apical papilla-derived cells were selected in this review. The characteristics (articles, clinical trial registration IDs, conditions, study designs, numbers of patients and cases, interventions, follow-up periods, main outcomes, and quality assessment) of these studies are summarized in Tables 1, 2, and 3 [15–34]. Funding sources of the included studies are listed in Supplementary Table 2.

**Table 1:** Summary of clinical trials using dental pulp- derived cells

References	Registration ID	Condition	Study Design	Patients, test/control	Interventions		Follow-up	Outcomes	Risk of Bias Assessments
					Test	Control			
D’aquino et al. [15]	NR	Socket preservation	SM-CT	17, 17/17	DPSCs + collagen sponge	Collagen sponge	3 months, 1 year	Radiography and clinical probing assessment revealed that optimal vertical repair and complete restoration of periodontal tissue were higher at the test site than the control site.	High
Brunelli et al. [16]	NR	Sinus floor elevation	A case report	1	Pulp micro-grafts + collagen sponge	–	4 months	Bone density in newly formed bone was about the double of native bone.	–
Aimetti et al. [17]	NR	Periodontal diseases	A case report	1	Pulp micro-grafts + collagen sponge	–	1 year	The defect was completely filled with bonelike tissue as confirmed through the reentry procedure.	–
Nakashima et al. [18]	NR	Irreversible pulpitis	Case series	5	DPSCs + G-CSF + atelocollagen	–	1, 2, 4, 12, 24 weeks	EPT demonstrated a robust positive response. MRI revealed that the regenerated tissue was similar to normal dental pulp.	–
Ferrarotti et al. [19]	NCT03386877	Periodontal diseases	RCT	29, 15/14	Pulp micro-grafts + collagen sponge	Collagen sponge	6 months, 12 months	Clinical and radiographic parameters revealed that test sites exhibited significantly more PD reduction, CAL gain, and bone defect fill than controls.	Low
Hernández-monjaraz et al. [20]	ISRCTN12831118	Periodontal diseases	A case report	1	Allogeneic DPSCs from deciduous teeth + collagen sponge	–	3 months, 6 months	The patient showed no sign of rejection and exhibited decreases in tooth mobility, PD and bone defect area.	–
Barbier et al. [21]	EudraCTdatabase 2014-001913-18	Socket preservation	SM-RCT	32, 32/32	Pulp micro-grafts + collagen matrix	Collagen matrix	6 months	No significant differences were found in the extent of bone repair during analyses of density or interdental septum height.	High
Xuan et al. [22]	NCT01814436	Dental pulp necrosis by trauma	RCT	40, 30/10	DPSCs aggregate from deciduous teeth	Apexification	12 months 24 months	Test group showed significantly higher improvement of EPT, vascular formation, root length, and width of the apical foramen.	High
Aimetti et al. [23]	NR	Periodontal diseases	Case series	11	Pulp micro-grafts + collagen sponge	–	6 months, 12 months	PD, CAL, and radiographic intrabony defect were improved.	–
NR [24]	NCT01932164	Cleft lip and palate	Case series	5	DPSCs from deciduous teeth + collagen + hydroxyapatite biomaterial	–	3 months, 6 months	Final completion of the alveolar defect with an 89.5% mean bone height was detected.	–

*NR* not reported, *SM* split-mouth, *CT* controlled trial, *G-CSF* granulocyte colony-stimulating factor, *EPT* electric pulp test, *MRI* magnetic resonance imaging, *CBCT* cone beam computed tomography, *RCT* randomized controlled trial, *PD* probing depth, *CAL* clinical attachment level

**Table 2:** Summary of clinical trials using periodontal ligament (PDL)-derived cells

References	Registration ID	Condition	Study design	Patients (teeth), test/control	Interventions		Follow-up	Outcomes	Risk of bias assessments
					Test	Control			
Feng et al. [25]	NR	Periodontal diseases	Case series	3 (16)	PDL progenitor + hydroxylapatite (Calcitite®)	–	3, 6, 12, 26, 32, 42, 72 months	PPD and CAL were decreased and gingival recession was increased.	–
Chen et al. 2016 [26]	NCT01357785	Periodontal diseases	RCT	30 (41), 20/21	PDLSC sheets + DBBM (Bio-oss®)	Bio-oss®	2 weeks, 3 months, 6 months, 1 year	No statistically significant differences were found for the increased CAL, PPD, or alveolar bone height between the test group and the control group.	Low
Iwata et al. [27]	UMIN000005027	Periodontal diseases	Case series	10 (10)	PDL-derived cell sheet + beta-tricalcium phosphate (β-TCP) granules	–	3 months, 6 months	PPD, CAL, and radiographic bone height were improved in all cases.	–

*NR* not reported, *RCT* randomized controlled trial, *PPD* periodontal probing depth, *CAL* clinical attachment level, *PDLSC* periodontal ligament stem cells, *DBBM* deproteinized bovine bone mineral

**Table 3:** Summary of clinical trials using gingiva- derived cells

References	Registration ID	Condition	Study design	Patients (sites), test/control	Interventions		Follow-up	Outcomes	Risk of bias assessments
					Test	Control			
Pini Prato et al. [28]	NR	Gingival augmentation	A case report	1 (1)	GF + benzyl ester of hyaluronic acid (HYAFF®)	–	1 months, 2 months, 3 months	A fully keratinized tissue was regenerated.	–
Pini Prato et al. [29]	NR	Gingival augmentation	Case series	6 (7)	GF + benzyl ester of hyaluronic acid	–	1 month, 3 months	An increased amount of gingiva was obtained, and the histological examination revealed a fully keratinized tissue on all the treated sites.	–
Mohammadi et al. [30]	NR	Insufficient attached gingiva	SM-RCT	9 (18), 9/9	GF + bovine skin collagen type I	Periosteal fenestration technique	3 months	The difference between the width of keratinized gingiva in test and control sites was significant.	Some concerns
Murata et al. [31]	NR	Gingival recessions	Case series	4 (14)	GF + atelo-collagen + hyaluronic acid sponge	–	13 to 40 weeks	The average root coverage and keratinized and attached gingival tissue were increased.	–
Jhaveri et al. [32]	NR	Gingival recessions	SM-RCT	10 (20), 10/10	GF + acellular dermal matrix allograft	Subepithelial connective tissue graft	3 months, 6 months	There were no significant differences between test and control sites for all measured clinical parameters.	Low
Köseoğlu et al. [33]	NR	Gingival recessions	SM-RCT	11 (22), 11/11	GF + collagen membrane	Collagen membrane	3 months, 6 months, 12 months	A statistically significant increase was detected in PRC in the test group compared with the control group.	Some concerns
Milinkovic et al. [34]	NR	Gingival recessions	SM-RCT	18 (48), 24/24	GF + collagen matrix (BioGide®)	Connective tissue graft	12 months	There was no statistically significant difference among groups regarding change in gingival recession coverage, CAL, and RES.	Some concerns

*NR* not reported, *SM* split-mouth, *RCT* randomized controlled trial, *GF* gingival fibroblasts, *PRC* percentage of root coverage, *CAL* clinical attachment level, *RES* root coverage esthetic score

Of the 10 selected clinical trials using dental pulp-derived cells, 4 were RCTs, 3 were case series, and 3 were case reports. Four studies were conducted for bone regeneration, and 2 studies were conducted for dental pulp regeneration. Although 4 studies were conducted for periodontitis treatment, one group published three clinical trials—a case report [17], a case series [23], and an RCT [19]—using the same materials. Nine clinical trials used autologous cells, and one used allogeneic cells [20]. Five studies used cultured cells derived from dental pulp tissue, and the remaining 5 studies used uncultured pulp micrografts. The dental pulp-derived cells used in 3 studies were isolated from deciduous teeth [20, 22, 24], whereas 7 studies used cells derived from permanent teeth. All trials except one [21] showed clinical benefits of cell transplantation therapies, including improvement of periodontal parameters, bone regeneration, and pulp regeneration. The split-mouth (SM) RCT for socket preservation after tooth extraction using pulp micrografts with a collagen matrix showed no significant differences in the extent of bone repair between the test group and the control group [21].

There were 3 clinical trials using periodontal ligament-derived cells. Of these, 2 were case series and one was an RCT. All studies were conducted for periodontal disease treatment. Two case series reported clinical effects, including improvement of periodontal parameters, whereas the RCT indicated no statistically significant differences between the test group and the control group [26].

Of the 7 studies that used transplanted gingiva-derived cells, 4 were SM-RCTs, 2 were case series, and one was a case report. One group published two clinical studies—a case report [28] and a case series [29]—that used gingival fibroblasts seeded onto the benzyl ester of a hyaluronic acid scaffold. All studies were conducted for gingival and soft tissue augmentation. Five studies reported the usefulness of gingiva-derived cell transplantation for gingival augmentation, whereas 2 RCTs showed no significant differences between the test and control sites [32, 34].

Risk of bias assessment showed that 3 of the 8 RCT included in this study were considered to have a low risk of bias, whereas 3 were assessed to have some concern of bias, and two were assessed to have a high risk of bias (Tables 1, 2, and 3). Details of the risk of bias assessment are presented in supplementary Table 3.

No adverse events that may have been associated with cell transplantation were reported in any study. Even clinical trials using allogeneic cells reported no signs of inflammation or symptoms of rejection [20]. This finding implied that cell-based regenerative therapy is a safe intervention.

## Discussion

Regenerative medicine with dental tissue-derived cells has been increasingly developed. This review indicated that dental pulp-derived cells, periodontal ligament-derived cells, and gingiva-derived cells showed some favorable clinical effects on tissue regeneration in patients. Moreover, no adverse events that may have been associated with cell transplantation were reported, suggesting the safety of dental tissue-derived cell-based therapy.

Dental pulp was the most frequently used cell source in clinical trials in this review. Dental pulp stem cells (DPSCs) were the first human dental tissue-derived MSCs to be identified from dental pulp tissue of permanent teeth and deciduous teeth [7, 35] and are widely studied due to their easy accessibility, noninvasive harvesting methods, and immunomodulatory properties, which would be suitable for preventing or treating T cell alloreactivity associated with hematopoietic or solid-organ allogeneic transplantation [36]. Half of the selected studies (5 studies) used cultured cells derived from dental pulp tissue. In these studies, DPSCs were applied for socket preservation and the treatment of irreversible pulpitis, periodontal diseases, dental pulp necrosis, and cleft lip and palate. The effectiveness of DPSC transplantation was confirmed in all clinical trials. These results suggested that the application of cultured DPSCs is clinically useful. We also had the good clinical results of bone regeneration for dental implants using cultured DPSCs. On the other hand, the remaining 5 studies used pulp micrografts, which require neither expansion nor manipulation of the cells in the procedure and has immediate application to clinical practice [16]. Some desired results were shown, but the SM-RCT for socket preservation after tooth extraction using pulp micrografts with a collagen matrix showed no significant differences in the extent of bone repair between the test and control groups [21]. The Rigenera method was used to produce adult MSCs from a minimum quantity of connective tissue of adult dental origin without culture. The reason that effective results were not obtained might be due to the process of producing transplants. It could be difficult to collect a sufficient number of stem cells and to confirm the contents.

Periodontal ligament-derived stem cells (PDLSCs) are multipotent stem cells that were first identified in 2004 [8]. PDLSCs were determined to have MSC-like properties, including self-renewal capacity, multipotency, in vivo tissue regeneration capacity, and immunomodulation [37]. PDLSCs can differentiate into periodontal ligament, alveolar bone, cementum, peripheral nerve, and vascular cells [38]. Preclinical animal studies indicated that PDLSC implantation could be expected to result in a beneficial outcome for periodontal regeneration [39]. This review revealed that all clinical trials using PDLSCs were conducted for periodontal disease treatment. Dental tissue-derived cells are good candidates for periodontal therapy because these cells are obtained from the periodontal ligament, dental pulp, gingiva, or bone in dental fields. In this review, two case reports and one RCT were selected. The case reports reported improvement of the periodontal index (periodontal probing depth, clinical attachment level, and radiographic bone height). On the other hand, the RCTs showed that using autologous PDLSCs to treat periodontal intrabony defects resulted in no significant differences in periodontal treatment outcomes between the cell-treated group and the control group. Although all three studies used periodontal ligament-derived cells, the cell processing procedure and the components of the transplanted products differed. It may be important for the clinical utilization of PDLSCs to establish proper methods to obtain and culture PDLSCs and generate effective scaffolds. Since only three clinical trials were completed, further studies are needed to assess the clinical usefulness of PDLSCs for treating periodontal diseases.

Connective tissue grafts have been applied for gingival recession or insufficiently attached gingiva to date. In traditional gingival and soft tissue augmentation procedures, an epithelial/connective tissue graft is used to cover the exposed periosteum to provide the necessary amount of keratinized tissue. However, tissue removal from the palate can cause considerable discomfort, pain, and morbidity to the patient during the harvesting procedure. On the other hand, in 2003, Pini Prato et al. [29] reported that in several cases, patients’ gingival fibroblasts were cultivated on a hyaluronic acid scaffold and were then implanted onto the exposed periosteum of the teeth where gingival augmentation was necessary. The tissue engineering technique of cultured gingival dermal substitute grafts composed of gingival fibroblasts and various matrices would contribute to an increase in root coverage and in keratinized and attached gingival tissue [29]. We also applied an injectable tissue engineering technique with MSCs, platelet-rich plasma, and hyaluronic acid to treat gingival recession (indicated by the black triangle) by injecting matrices. This method is minimally invasive, and long-term esthetic improvement was achieved [40]. Therefore, cell-based therapies might be considered a new predictable and effective alternative procedure for gingival and soft tissue augmentation.

In addition, this study revealed that periodontal ligament-derived cells and gingival-derived cells were applied only for regeneration of their corresponding tissue. However, dental pulp-derived cells were useful not only for regeneration of dental pulp but also of other tissues, including bone and periodontal tissue, in clinical trials. The previous basic and preclinical animal studies also supported the evidence that DPSCs are useful for bone and periodontal regeneration [41–43].

In this systematic review, only the clinical trials that were completed and had complete results were included. According to the electronic search of ClinicalTrials.gov and CENTRAL, there are several ongoing clinical trials using dental tissue-derived cells. Since the results of basic and preclinical studies have indicated that DPSCs have potential for effective application for several kinds of systemic diseases [44], clinical trials using DPSCs for systemic diseases such as diabetes (NCT03912480), knee osteoarthritis (NCT04130100), and liver cirrhosis (NCT03957655) have also been conducted. On the other hand, the clinical trials and clinical application of MSCs are still controversial because of the large gap between basic and translational research and the strict regulations.

In the future, dental tissue-derived cells will be used more frequently because they can be obtained in an easy and minimally invasive manner [45]. These new sources of cells could be beneficial for cellular therapy and advancing the development of regenerative medicine strategies, such as allogeneic transplantation or therapy with extracellular vesicles (EVs), which are nanoscale membrane vesicles including exosomes actively released by cells [46]. EVs have also been used directly or engineered as therapeutic agents with multiple applications, such as regenerative medicine [47], cancer therapy [48], and immunomodulation [49]. Therefore, dental tissue-derived cell-based regenerative medicine could provide insights into emerging potential expansion and novel therapeutic approaches to induce endogenous modulation (e.g., immune activation) or exogenous (e.g., drug- and protein-mediated) effects [46] and could be applicable in various fields.

More clinical trials should be conducted to evaluate the effectiveness of cell-based therapy. In addition, the issue of cost-effectiveness also would be taken into consideration. Taken together, these findings evolve and indicate that cell-based regenerative therapy using dental tissue-derived cells would be a promising tool for the treatment of various diseases and will proceed to use in the development of medicine in the future.

## Supplementary information

**Additional file 1.**

**Additional file 2.**

**Additional file 3.**

## Data Availability

Not applicable.

## Abbreviations

ES: Embryonic stem
iPS: Induced pluripotent stem
MSCs: Mesenchymal stem cells
RCTs: Randomized controlled trials
CTs: Controlled trials
SM: Split-mouth
DPSCs: Dental pulp stem cells
PDLSCs: Periodontal ligament-derived cells
