# Supplementary material for: Promising advances in clinical trials of dental tissue-derived cell-based regenerative medicine
Source: Stem Cell Res Ther. 2020 May 12;11:175. doi: 10.1186/s13287-020-01683-x (PMC7218566; doi:10.1186/s13287-020-01683-x)
Supplement: Supplementary file 1 — Additional file 1. [file 13287_2020_1683_MOESM1_ESM.docx]

Supplementary Table 1. List of studies excluded by full-text search.

|  | Registration ID | Title | Reasons | References |
| --- | --- | --- | --- | --- |
| **dental pulp- derived cells** | NCT03102879 | Encapsulated Mesenchymal Stem Cells for Dental Pulp Regeneration. | Not related | - |
|  | NCT03171194 | Pilot Trial of Mesenchymal Stem Cells for Systemic Lupus Erythematosus | Not related | - |
|  | NCT03766217 | Bone Tissue Engineering with Dental Pulp Stem Cells for Alveolar Cleft Repair | Incomplete | - |
|  | JPRN-UMIN000016515 | A Clinical Study of Bone Regeneration Using Autologous Dental Pulp Stem Cells | No Results Available | - |
|  | - | TOOTH (The Open study of dental pulp stem cell Therapy in Humans): Study protocol for evaluating safety and feasibility of autologous human adult dental pulp stem cell therapy in patients with chronic disability after stroke. | Incomplete | Int J Stroke. 2016;11(5):575-85. doi: 10.1177/1747493016641111. |
|  | - | PERSPECTIVES: Stroke survivors' views on the design of an early-phase cell therapy trial for patients with chronic ischaemic stroke. | Incomplete | Health Expect. 2019;22(5):1069-1077. doi: 10.1111/hex.12932. |
| **periodontal ligament -derived cells** | NCT01082822 | Periodontal Ligament Stem Cell Implantation in the Treatment of Periodontitis | No Results Available | - |
|  | ISRCTN13093912 | Cell Therapy in Periodontal Regeneration. A Double-Blinded Randomized Parallel Groups controlled clinical trial | No Results Available | - |
| **gingiva- derived cells** | NCT03570333 | Progenitor Potential of Mesenchymal Stem Cells in Palatal Tissue Harvested From Molar and Premolar Sites | Incomplete | - |
|  | NCT03638154 | Regenerative Potential of Cultured Gingival Fibroblast- Mesenchymal Stem Cells in Treatment of Periodontitis | No Results Available | - |
