# Supplementary material for: Promising advances in clinical trials of dental tissue-derived cell-based regenerative medicine
Source: Stem Cell Res Ther. 2020 May 12;11:175. doi: 10.1186/s13287-020-01683-x (PMC7218566; doi:10.1186/s13287-020-01683-x)
Supplement: Supplementary file 2 — Additional file 2. [file 13287_2020_1683_MOESM2_ESM.docx]

Supplementary Table 2. Funding sources of the included studies.

|  | References | Registration ID | Funding Sources |
| --- | --- | --- | --- |
| **Dental pulp- derived cells** | D'aquino et al. 2009 [15] | NR | Italian MIUR |
|  | Brunelli et al. 2013 [16] | NR | University, PRIN 2008 and Regione Emilia Romagna |
|  | Aimetti et al. 2014 [17] | NR | University |
|  | Nakashima et al. 2017 [18] | NR | Budget for promoting science and technology in Japan |
|  | Ferrarotti et al. 2018 [19] | NCT03386877 | University |
|  | Hernández-monjaraz et al. 2018 [20] | ISRCTN12831118 | General Directorate of Academic Personnel Affairs and University |
|  | Barbier et al. 2018 [21] | EudraCTdatabase 2014-001913-18 | University, PRIN 2008 and Regione Emilia Romagna |
|  | Xuan et al. 2018 [22] | NCT01814436 | National Key Research and Development Program of China, the Nature Science Foundation of China, and a Schoenleber pilot grant from the University of Pennsylvania  School of Dental Medicine. |
|  | Aimetti et al. 2018 [23] | NR | University |
|  | NR [24] | NCT01932164 | University |
| **Periodontal ligament -derived cells** | Feng et al. 2010 [25] | NR | California Institute for Regenerative Medicine and Forward Dentist Group, Taiwan |
|  | Chen et al. 2016 [26] | NCT01357785 | University |
|  | Iwata et al. 2018 [27] | UMIN000005027 | Ministry of Education, Culture, Sports, Science and Technology, Japan |
| **Gingiva- derived cells** | Pini Prato et al. 2000 [28] | NR | NR |
|  | Pini Prato et al. 2003 [29] | NR | NR |
|  | Mohammadi et al. 2007 [30] | NR | Pasteur Institute of Iran and University |
|  | Murata et al. 2008 [31] | NR | Fund for Scientific Promotion of Tanaka Industries Co., Ltd., Niigata, and Grant-in-Aid for Scientific Research (B) from the Japan Society for the promotion of Science |
|  | Jhaveri et al. 2009 [32] | NR | The International Clinical Dental Research Organization, Pune, India |
|  | Köseoğlu et al. 2013 [33] | NR | Scientific and Technological Research Council of Turkey |
|  | Milinkovic et al. 2015 [34] | NR | Ministry of Education and Science, Republic of Serbia |

NR, not reported
