# Supplementary material for: Promising advances in clinical trials of dental tissue-derived cell-based regenerative medicine
Source: Stem Cell Res Ther. 2020 May 12;11:175. doi: 10.1186/s13287-020-01683-x (PMC7218566; doi:10.1186/s13287-020-01683-x)
Supplement: Supplementary file 3 — Additional file 3. [file 13287_2020_1683_MOESM3_ESM.docx]

Supplementary Table 3. Risk of bias assessment using Revised Cochrane risk-of bias tool for randomized trials (RoB 2).

|  | Domain 1 | Domain 2 | | Domain 3 | Domain 4 | Domain 5 | Overall |
| --- | --- | --- | --- | --- | --- | --- | --- |
|  | **Randomisation** | **Assignment** | **Adherence** | **Missing Outcome Data** | **Outcome Measurement** | **Selection of Reported Outcome** |  |
| Ferrarotti et al. 2018 | Low | Low | Low | Low | Low | Low | Low |
| Barbier et al. 2018 | Low | High | Low | Low | Low | Low | High |
| Xuan et al. 2018 | Low | High | Low | Low | Low | Low | High |
| Chen et al. 2016 | Low | Low | Low | Low | Low | Low | Low |
| Mohammadi et al. 2007 | Some Concerns | Some Concerns | Low | Low | Low | Low | Some Concerns |
| Jhaveri et al. 2009 | Low | Low | Low | Low | Low | Low | Low |
| Köseoğlu et al. 2013 | Low | Some Concerns | Low | Low | Some Concerns | Low | Some Concerns |
| Milinkovic et al. 2015 | Low | Some Concerns | Low | Low | Low | Low | Some Concerns |
